# Molecularly Imprinted Silica-Coated CdTe Quantum Dots for Fluorometric Determination of Trace Chloramphenicol

**DOI:** 10.3390/molecules26195965

**Published:** 2021-10-01

**Authors:** Xiaoxiao Chen, Yang Liu, Pu Li, Yichen Xing, Chaobiao Huang

**Affiliations:** 1 College of Chemistry and Life Science, Zhejiang Normal University, Jinhua 321004, China; 26947255692@zjnu.edu.cn (X.C.); 202120200767@zjnu.edu.cn (Y.L.); 202020200588@zjnu.edu.cn (P.L.); yanlieyi37485nd@126.com (Y.X.); 2 Xingzhi College, Zhejiang Normal University, Jinhua 321004, China

**Keywords:** molecular imprinting, chloramphenicol, quantum dots, fluorescence quenching

## Abstract

A dual recognition system with a fluorescence quenching of quantum dots (QDs) and specific recognition of molecularly imprinted polymer (MIP) for the detection of chloramphenicol (CAP) was constructed. MIP@SiO_2_@QDs was prepared by reverse microemulsion method with 3-aminopropyltriethoxysilane (APTS), tetraethyl orthosilicate (TEOS) and QDs being used as the functional monomer, cross-linker and signal sources, respectively. MIP can specifically recognize CAP, and the fluorescence of QDs can be quenched by CAP due to the photo-induced electron transfer reaction between CAP and QDs. Thus, a method for the trace detection of CAP based on MIP@SiO_2_@QDs fluorescence quenching was established. The fluorescence quenching efficiency of MIP@SiO_2_@QDs displayed a desirable linear response to the concentration of CAP in the range of 1.00~4.00 × 10^2^ μmol × L^−1^, and the limit of detection was 0.35 μmol × L^−1^ (3σ, *n* = 9). Importantly, MIP@SiO_2_@QDs presented good detection selectivity owing to specific recognition for CAP, and was successfully applied to quantify CAP in lake water with the recovery ranging 102.0~104.0%, suggesting this method has the promising potential for the on-site detection of CAP in environmental waters.

## 1. Introduction

Chloramphenicol is a broad-spectrum antibiotic which is widely used in the treatment of infectious diseases of aquatic animals, livestock and poultry animals, as well as a growth promoter [1]. However, it is strongly toxic and has side effects which can cause symptoms such as the bone marrow suppression of aplastic anemia, the hypoplasia of platelets and leukopenia [2]. To minimize possible exposure to antibiotics, CAP was prohibited in food-producing animals in the U.S. and is listed the European Union’s “group A” list. Unfortunately, due to the low cost of CAP production, it is still used illegally in less developed areas. Some studies have pointed out that CAP is difficult to completely metabolize and a large percentage of the primary drugs are discharged into the aquatic environment [3]. Therefore, CAP can be detected in environmental waters [4,5,6]. Various methods of CAP detection have been reported, such as chromatography [7,8], chemiluminescent [9,10], enzyme-linked immunosorbent assay [11], electrochemical analysis [12,13] and aptamer sensor [14]. Although each of these methods has its own advantages, many of these have several disadvantages, such as their high detection cost, cumbersome sample pretreatment and complicated operation procedures. These limitations prevent these techniques from being used for the rapid in situ detection of CAP. Therefore, it is of great practical importance to establish a simple, cost-effective, rapid and highly selective analytical method for CAP that can be expected to be used in field detection.

Molecular imprinting technology (MIT) is known as a technology that uses template memory to form selective sites in the MIP matrix [15]. MIPs have drawn more attention [16] because of their high physicochemical robustness, ease of preparation and specific recognition of target molecules. As receptors with antibody-like binding properties or enzyme-like activity, MIPs are best described as synthetic analogues to the natural, biological antibody–antigen systems and have gradually become the preferred choice for molecular receptor materials [15,17,18]. Their preparation involves the polymerization of appropriate monomers and cross-linkers in the presence of selected template molecules. Upon the removal of template molecules, recognition sites of similar shape and size and complementary chemical function are generated in the highly cross-linked polymer matrix [19,20]. Subsequently, target molecules interact with recognition sites in a covalent or non-covalent manner and reconnect into template pores. Compared to the covalent method, the non-covalent binding method has wider application prospects [21]. The resultant MIPs have shown their great potential in various fields, such as chromatographic separation [22], solid phase extraction [23], simulated enzyme catalysis [24], electrochemical sensors [25] and optical sensors [26].

Quantum dots have become an emerging light-emitting material with a unique size effect and luminescence properties [27,28,29]. CdTe QDs, as one among semiconductor nanoparticles, have shown great potential for signal transduction in chemical sensors because of their good photostability and high luminescence efficiency [30]. Compared with traditional organic fluorescent dyes, these have the advantages of a wide absorption band, narrow emission bandwidth and excellent optical stability. However, their lack of selectivity is a key factor hindering their application as a powerful detection tool [31,32]. In order to improve the selectivity of QDs, efforts have been devoted to improve the development of the surface modification technology of QDs [33,34,35]. Among these, MIT is a powerful means of improving the surface selectivity of QDs while ensuring their luminescence properties [36,37,38]. Through the action of a cross-linker and initiator, QDs are polymerized with a functional monomer and template molecule (target analyte) to form MIP@QDs. After removing the template molecule (target analyte), the cavities inside MIP@QDs have the specificity to recognize the target analyte [39]. Once target analyte appears, it will be specifically identified by MIP@QDs. Thus, the introduction of MIP can effectively improve the selectivity of QDs. In this case, it is necessary to obtain MIPs with their wonderful binding property, and the key to this lies in the controllability and hydrophilicity of the imprinted coating. To date, several monomers have been used for the preparation of imprinting coatings, such as dopamine, aniline, acrylamide and siloxane [40]. Silica-coated QDs possess excellent hydrophilicity, controllability and fluorescence stability owing to the easy functionalization of amino and carboxyl groups on their surface [33].

**Scheme 1:**
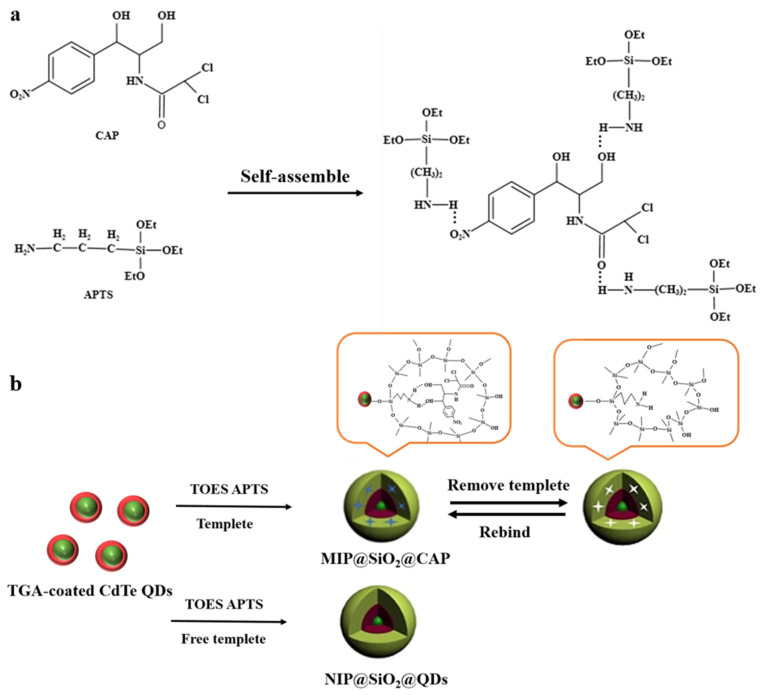
Schematic procedure for the preparation of MIP@SiO_2_@CAP (**a**) and the synthesis routes of MIP@SiO_2_@QDs and NIP@SiO_2_@QDs (**b**).

Inspired by these studies, we propose a dual recognition system with the fluorescence quenching of QDs and specific recognition of MIP for CAP detection. As shown in Scheme 1a, the synthesis of MIP@SiO_2_@QDs is provided by reverse microemulsion method and a surface imprinting technique. With 3-aminopropyltriethoxysilane (APTS) as the functional monomer, CAP was polymerized by spontaneous hydrogen bond assembly. Then, as shown in Scheme 1b, under the action of catalyst NH_3_·H_2_O and cross-linking agent TEOS, CdTe QDs were embedded in the layer of SiO_2_. At the same time, CAP was imprinted onto the surface of SiO_2_@QDs by the binding sites of APTS-NH_2_ provided by TEOS, and then MIP@SiO_2_@QDs was obtained. The coating of SiO_2_ shells improves the photochemical stability of QDs, while preventing the leaching of toxic metals from QDs and reducing secondary pollution to the environment. After the removal of CAP, specific recognition sites that are similar in shape and size and have complementary chemical functions were formed in the silica matrix. Finally, CAP detection was achieved by the specific recognition of MIP and the fluorescence burst of QDs. Furthermore, this method was applied to rapidly respond to CAP in lake water.

## 2. Result and Discussion

### 2.1. Characterization

**Figure 1:**
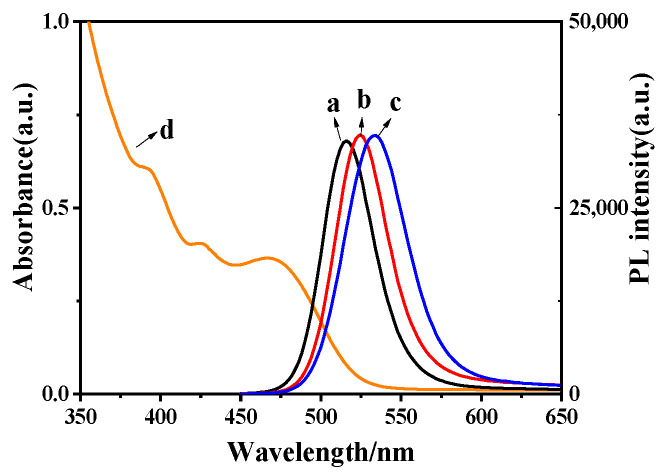
Fluorescence and absorption spectrum of CdTe QDs (curves **a**, **b** and **c**: fluorescence emission peak; curve **d**: ultraviolet absorption peak—the reflux times of curves **a**, **b** and **c** are 2 h, 4 h and 6 h, respectively).

Figure 1 displays the ultraviolet absorption and fluorescence emission spectra of CdTe QDs. Among them, curves a, b and c represent the fluorescence emission spectra of CdTe QDs with increasing reflux time. The fluorescence emission peak is near 525 nm and shows a tendency of red shift, while its half-peak width gradually increases, which is attributed to the increased particle size and the wider particle size distribution of QDs. In addition, curve d reveals that the absorption boundary of QDs is approximately 550 nm.

**Figure 2:**
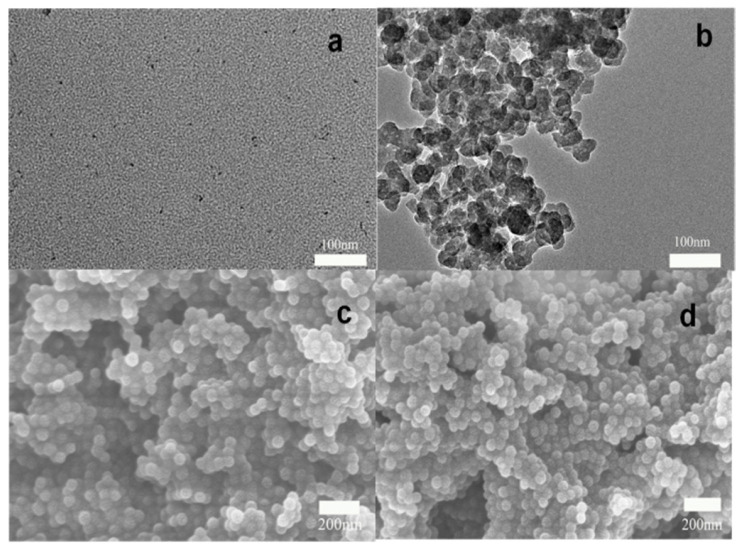
TEM images of CdTe QDs (**a**) and MIP@SiO_2_@QDs (**b**), SEM images of NIP@SiO_2_@QDs (**c**) and MIP@SiO_2_@QDs (**d**).

A transmission electron microscope (TEM) was used to determine the morphology of CdTe QDs, MIP@SiO_2_@QDs and NIP@SiO_2_@QDs. As shown in Figure 2a, the well-dispersed CdTe QDs with an average size of 3 nm basically appeared regularly spherical. As we can see in Figure 2b, the particle size of MIP@SiO_2_@QDs was significantly increased to 40~60 nm. The corresponding SEM images of MIP@SiO_2_@QDs and NIP@SiO_2_@QDs are presented in Figure 2c,d. Both of these, in the particle size range of 40~60 nm, are fairly uniformly spherical.

**Figure 3:**
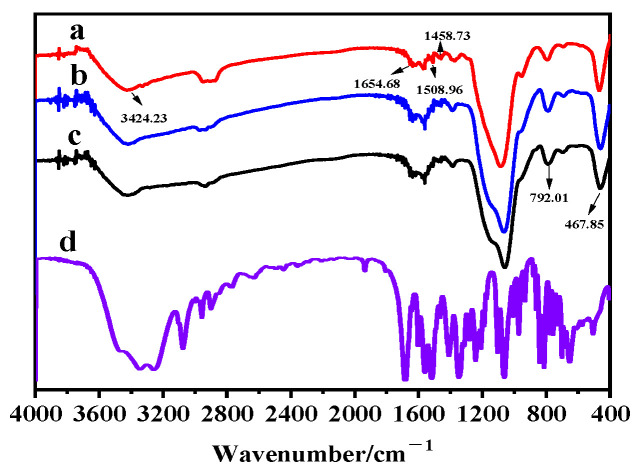
FT-IR spectra of MIP@SiO_2_@CAP (**a**), MIP@SiO_2_@QDs (**b**), NIP@SiO_2_@QDs (**c**) and CAP (**d**).

The FT-IR spectra of MIP@SiO_2_@CAP, MIP@SiO_2_@QDs, and NIP@SiO_2_@QDs are given in Figure 3. The characteristic peak of the Si–O–Si [27] stretching vibration is a wide peak of 1000~1100 cm^−1^, the asymmetric tensile vibration peaks of Si-O are 467.85 cm^−1^ and 792.01 cm^−1^ and the stretching vibration peaks of the amino group are 3424.23 cm^−1^ and 1654.68 cm^−1^ [27], indicating that CAP has successfully been grafted on the surface of SiO_2_@QDs. In the infrared spectrum of CAP (Figure 3d), the characteristic absorption peaks at 1458.73 and 1508.96 cm^−1^ which are attributed to the stretching vibration of aromatic ring in CAP molecule [41] can be observed. However, as shown in Figure 3b,c, the absorption peaks at 1458.73 cm^−1^ and 1508.96 cm^−1^ [32] are significantly different from those in Figure 3a, suggesting that CAP was effectively eluted.

**Figure 4:**
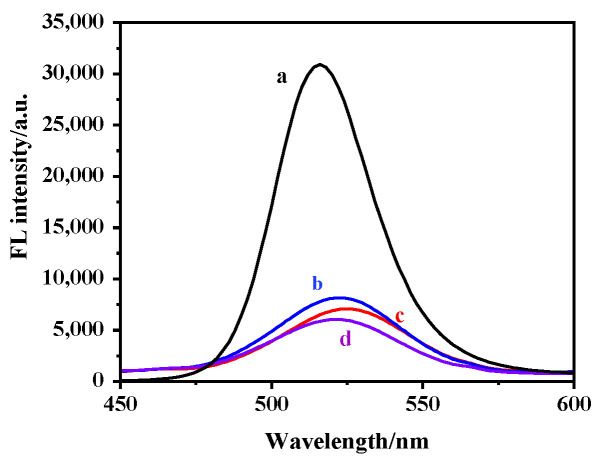
Fluorescence spectra of the CdTe QDs (**a**), MIP@SiO_2_@QDs (**b**), NIP@SiO_2_@QDs (**c**) and MIP@SiO_2_@CAP (**d**) (the added concentration of CAP in MIP@SiO_2_@CAP was 50.00 μmol·L^−1^).

In addition, the fluorescence emission spectrum greatly demonstrates that CAP has participated in SiO_2_@QDs. As shown in Figure 4, the fluorescence emission spectrum of MIP@SiO_2_@QDs showed a slightly red shift compared to CdTe QDs, which was based on the fact that the porous nature of the silica layer provided a sufficiently open pathway for diffusion and interaction with the core of small molecules, and thus small molecules in the solution diffused into the silica layer and interacted with the core of QDs, resulting in the passivation of the surface of the QDs and the red-shifting of the fluorescence emission peak [41]. In addition, compared with MIP@SiO_2_@CAP, the fluorescence emission intensity of the MIP@SiO_2_@QDs was obviously higher. The above results indicate that the MIP@SiO_2_@QDs was successfully prepared.

### 2.2. Adsorptive Property of MIP@SiO_2_@QDs

**Figure 5:**
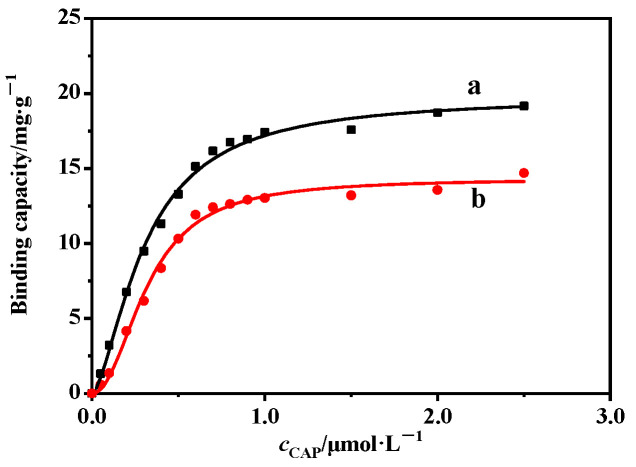
Isothermal adsorption of MIP@SiO_2_@QDs (**a**) and NIP@SiO_2_@QDs (**b**).

In order to evaluate the adsorptive property of MIP@SiO_2_@QDs for CAP, equal amounts of MIP@SiO_2_@QDs and NIP@SiO_2_@QDs were mixed with different concentrations of CAP for isothermal adsorption experiments. According to Figure 5, we can find that the adsorption capacity of the polymer gradually increased with the increase in the concentration of CAP and eventually tended towards the ultimate saturation. The maximum adsorption capacity of MIP@SiO_2_@QDs was larger than that of NIP@ SiO_2_@QDs. This was due to the presence of a large number of cavities in the silica layer of MIP@SiO_2_@QDs matching the size and shape of CAP which enhanced its adsorption capacity. Previously, Chen et al. [42] reported that the maximum adsorption value (Q_m_) of a magnetic molecularly imprinted polymer for CAP was 2.32 mg·g^−1^. Dai et al. [3] reported that the value of Q_m_ was 4.85 mg·g^−1^. Here, the Q_m_ value of our synthesized MIP@SiO_2_@QD was 18.89 mg·g^−1^, which exhibited a higher adsorption performance.

### 2.3. Optimization of Elution Times

**Figure 6:**
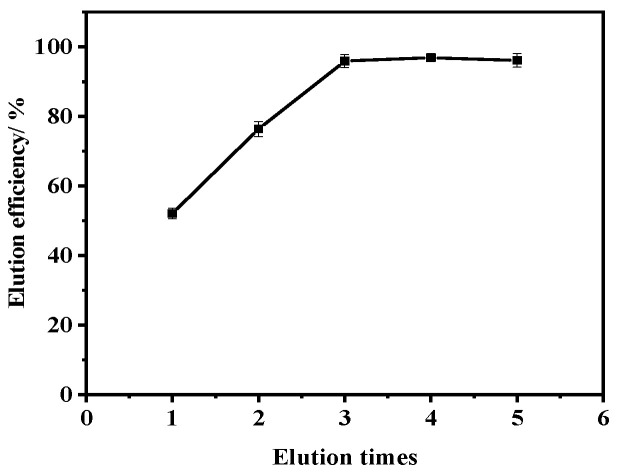
Elution efficiency of the different number of elution times (each elution operation was performed for 10 min).

To achieve the best elution efficiency, elution times (the number of repetitions of the elution operation) was systematically investigated and optimized. According to the image of elution efficiency (Figure 6), after three repetitions, the template molecule of CAP was basically eluted. Therefore, three times was taken as the optimal condition of elution times.

### 2.4. Effect of pH on Fluorescence of MIP@SiO_2_@QDs

**Figure 7:**
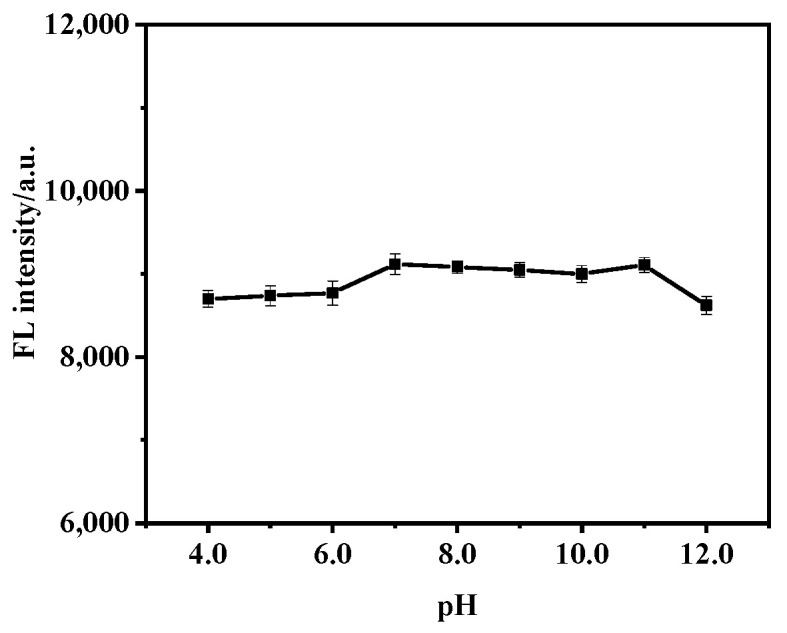
Effect of pH on the fluorescence of MIP@SiO_2_@QDs (taking the pH of 8.0 as the suitable condition for subsequent research).

The fluorescence detection condition of pH was also optimized. Figure 7 shows the fluorescence trend of MIP@SiO_2_@QDs in the pH range of 4.0~12.0. The fluorescence intensity of MIP@SiO_2_@QDs was relatively stable in the pH range of 4.0~11.0, which indicated that the silica coating provided QDs with certain acid and alkali resistance. This is probably because under acidic conditions, hydrogen ions destroyed the binding of cadmium ions (Cd^2+^) to hydroxyl groups, allowing the unsaturated Cd^2+^ in the TGA-Cd complex to combine with Te and form a TGA-Cd shell on the surface of CdTe QDs [27], which made up the surface defects and effectively improved the fluorescence intensity. When the pH exceeded 11.0, the fluorescence intensity decreased, which might because silicon hydroxyl groups were ionized in a strong alkali environment and attacked the surface of QDs in a nucleophilic way to produce defects—resulting in decreased fluorescence [33]. Compared to molecularly imprinted polymers reported in other literatures [28,33,43], our synthesized MIP@SiO_2_@QDs had better acid and alkaline resistance. For example, Zhou et al. [33] reported the pH stability range of MIP as 4.0~8.0. Ensafi and co-workers [43] reported that the prepared molecularly imprinted polymer remained stable within the pH range of 7.0~9.0. The improvement of the acid and alkali resistance of MIPs can further simplify the pretreatment of samples and enhance their applicability.

### 2.5. Sensitivity of MIP@SiO_2_@QDs

**Figure 8:**
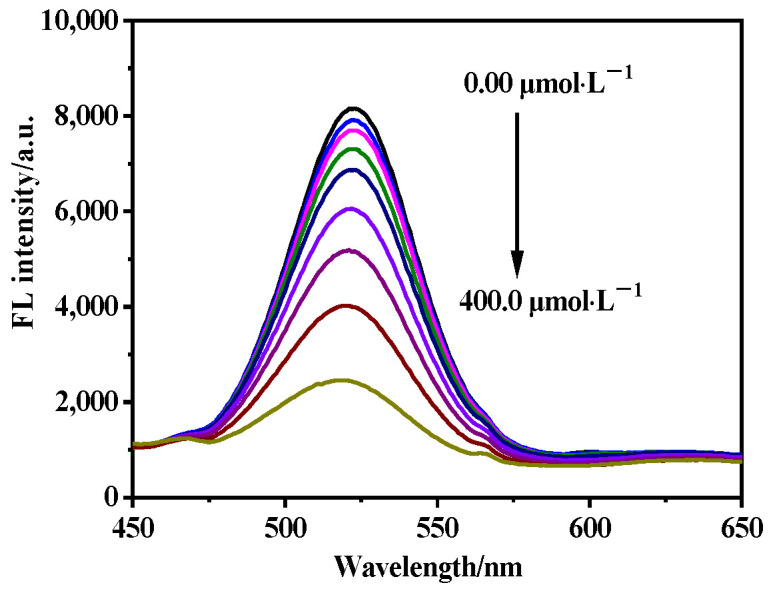
Fluorescence spectra of the MIP@SiO_2_@QDs with different concentrations of CAP.

The detection performance of MIP@SiO_2_@QDs for CAP was further studied under the optimal conditions. As seen from Figure 8, the fluorescence intensity of MIP@SiO_2_@QDs gradually decreased with the increase in CAP concentration, and the relationship between the concentration of the quenching agent CAP and the fluorescence intensity satisfied the Stern-Volmer equation:

(1) $$F_{0}/F\;=1+k_{sv}c$$

where F_0_ and F are the fluorescence intensity of MIP@SiO_2_@QDs in the absence and presence of CAP, respectively. *k_sv_* is the quenching constant of the Stern–Volmer equation, and *c* is the concentration of CAP.

**Figure 9:**
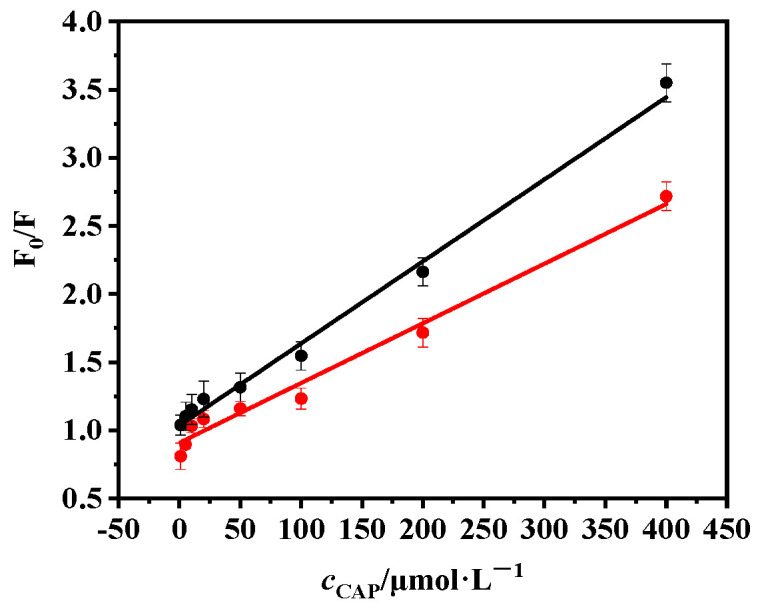
Stern-Volmer plots from MIP@SiO_2_@QDs (**a**) and NIP@SiO_2_@QDs (**b**) with CAP.

Figure 9 shows the Stern-Volmer plots from MIP@SiO_2_@QDs and NIP@SiO_2_@QDs with CAP. For MIP@SiO_2_@QDs, F_0_/F showed a good linear relationship with the concentration of CAP in the range of 1.00~4.00 × 10^2^ μmol·L^−1^. The linear equation was F_0_/F = 0.0061*c* + 1.027 (R^2^ = 0.9973) and the limit of detection (LOD) was 0.35 μmol·L^−1^ (3σ, *n* = 9). For NIP@SiO_2_@QDs, the correlation coefficient was 0.9820 and the linear regression equation was F_0_/F = 0.0044*c* + 0.9095. It is obvious that MIP@SiO_2_@QDs performed a more obvious response to CAP than NIP@SiO_2_@QDs due to its fluorescence quenching amount being more apparent.

### 2.6. Selectivity of MIP@SiO_2_@QDs

**Figure 10:**
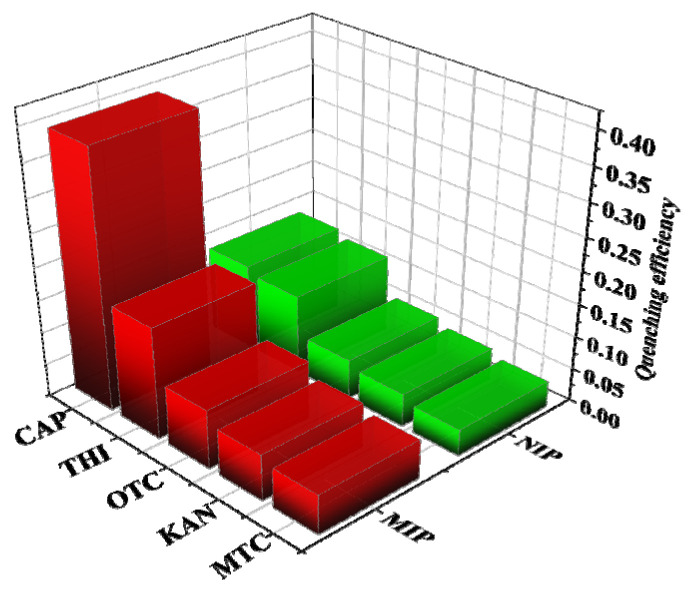
Quenching efficiency of MIP@SiO_2_@QDs and NIP@SiO_2_@QDs (the concentration of CAP, THI, OTC, KAN and MTC is 100.0 μmol·L^−1^_,_ respectively).

To investigate the selective recognition ability of MIP@SiO_2_@QDs, THI, OTC, KAN and MTC were selected as structural analogues and researched at the same concentrations. The results are shown in Figure 10. It is clear that CAP has the highest fluorescence burst efficiency for MIP@SiO_2_@QDs. NIP@SiO_2_@QDs has a certain response, however, the quenching level is relatively close and there is no selectivity. Since these analogues struggled to match the imprinted cavity, only the template molecule of CAP was adapted to the imprinted cavity in terms of shape, size and functional groups, suggesting that MIP@SiO_2_@QDs had a highly selectivity for CAP.

### 2.7. Real Sample Detection

In order to verify the effectiveness and applicability of this method, we selected lake water which was taken from the lake of Zhejiang Normal University as the real sample. The water sample was removed from the suspended solids by 0.22 μm filter membrane, and then the filtrate was used for fluorescence detection. It was found that the water sample was no nonentity of CAP, which was further confirmed by the absence of a UV characteristic absorption at 278 nm. Then, a series of concentrations of CAP (10.00, 85.00, 150.0 and 300.0 μmol·L^−1^) was added to the water sample by sample preparation, and spiked recovery experiments were carried out. Results (Table 1) showed that the CAP concentration quantified by MIP@SiO_2_@QDs was in general agreement with that actually added and the recovery rate was calculated as in the range of 100.2~104.0%. In addition, the reproducibility for MIP@SiO_2_@QDs was tested by performing 10 replicate measurements in 100.0 μmol·L^−1^ of CAP solution. As a result, the relative standard deviation (RSD) is 2.06%. These results suggest that this method has the promising potential for the detection of CAP in lake water. 

The performance of the prepared MIP@SiO_2_@QDs was contrasted with many other analytical methods for CAP that were previously reported (Table 2). Compared to other analytical methods, this method is comparable or better than them. Compared with HPLC and ELISA, the LOD of this method is slightly worse, but this method has a larger linear range and better reproducibility. In addition, the ELISA and aptamer sensor both involve the assembly of nucleic acid aptamers or enzymes and the specific antigen–antibody binding, which are particularly susceptible to environmental conditions and have a relatively high cost. This limits the application of these methods in field testing. MIP@SiO_2_@QDs has excellent stability and is not easily affected by the environment. Hence, this method has a higher utility for the in situ detection of CAP.

## 3. Experiment

### 3.1. Material and Reagents

Chloramphenicol (CAP), oxytetracycline (OTC), kanamycin (KAN), metacycline (MTC), thiamphenicol (THI), tetraethoxysilane (TEOS), tellurium powder (TE) and 3-aminopropyltriethoxysilane (APTS) were purchased from Aladdin (Shanghai, China). Mercaptoacetic acid (TGA) was acquired from Sigma-Aldrich (Saint louis, MO, USA). 1-hexanol and CdCl_2_·2.5H_2_O were obtained from Sinopharm Chemical Reagent (Zhejiang, China). NH_3_·H_2_O was supplied by Lanxi Sanfeng Chemical (Zhejiang, China). The acetic acid–sodium acetate buffer solution (0.1 mol × L^−1^, pH 4.0~5.0), the phosphate-buffered solution (0.1 mol × L^−1^, pH 6.0~10.0) and the sodium carbonate-sodium bicarbonate buffer solution (0.1 mol × L^−1^, pH 11.0~12.0) were prepared. The water used in the experiment was freshly prepared ultrapure water.

### 3.2. Instruments

The morphological evaluation was characterized by JEM-2100F transmission electron microscopy (TEM) and the S-4800 Scanning Electron Microscope (SEM). The FT-IR spectra were recorded on a Nexus 670 infrared spectrometer (Reonicolai Inc., New York, NY, USA). The UV-vis absorption spectrum was obtained with a Lambda 950 spectrophotometer (PerkinElmer Inc., Waltham, MA, USA). Fluorescence (FL) spectra were recorded using the RF-6000 Fluorescence Spectrometer (Shimadzu, Kyoto, Japan). 

### 3.3. Synthesis of TGA-Stabilized CdTe QDs

Preparation of TGA-stabilized CdTe QDs was modified as previously reported [38]. Tellurium powder (0.04 g, 0.3 mmol), NaBH_4_ (0.1 g, 2.7 mmol) and 12 mL ultrapure water were successively added into a three-necked flask. The mixture was stirred on a 0 °C ice bath for 4 h in the condition of N_2_ flow to give the aqueous solution of NaHTe.

CdCl_2_·2.5H_2_O (0.7 g, 0.3 mmol), 60 μL TGA and 50 mL of ultrapure water were added to a 100 mL three-necked flask. The pH of the mixture was set to 8.0 using NaOH solution (0.1 mol × L^−1^) and stirred in N_2_ flow for 30 min. Then, 6 mL freshly prepared NaHTe precursor solution was quickly injected (the molar ratio of Cd: Te: TGA was approximately 2:1:4.8) and the mixture was heated and refluxed for 2 h, 4 h and 6 h. Finally, after the treatment of the precipitation with ethanol, washing and redispersion, CdTe QDs (2.0 mg·mL^−1^) were collected and kept at 4 °C for further use.

### 3.4. Synthesis of MIP@SiO_2_@QDs and NIP@SiO_2_@QDs

MIP@SiO_2_@QDs was synthesized basing on the modified reverse microemulsion method as previously reported in [45]. Into a three-necked bottle, 1.75 mL cyclohexane, 1.75 mL Triton X-100 and 7.5 mL 1-hexanol were added and stirred at room temperature for 20 min. Then, CdTe QDs (1 mL, 2.0 mg×mL^−1^) were added into the mixture with vigorous stirring. After 15 min, 100 μL TEOS and 60 μL NH_3_·H_2_O were mixed in the solution for stirring 2 h. Then, adding 20 μL APTS hexanol solution and 5.0 mg CAP for stirring 24 h. The resulting mixture was demulsified with acetone, and after being centrifuged at 4000 rpm for 5 min, the supernatant was ruled out to collect precipitate. Finally, the precipitate was washed with a mixed solvent of ethanol and acetonitrile (*v/v*, 8:2) to remove CAP and other impurities. This operation was repeated three times. After being baked at 60 °C for 24 h, MIP@SiO_2_@QDs was obtained. The preparation of NIP@SiO_2_@QDs was similar to that of MIP@SiO_2_@QDs, but CAP was absent. 

### 3.5. Adsorption Experiment

Five milligrams (5.0 mg) of MIP@SiO_2_@QDs and NIP@SiO_2_@QDs was successively added into different concentrations of CAP standard solutions, respectively, and then shaken for 2 h. After placing for 5 h, the mixtures were centrifuged and filtered with a filter membrane (0.22 μm) for UV–vis spectrophotometer analysis. The adsorption capacity of MIP@SiO_2_@QDs and NIP@SiO_2_@QDs for CAP was quantified by the following equation:

(2) $$Q=\frac{M\times V\times (c_{1}-c_{2})}{m}$$

where *Q* (μg×g^−1^) represents the adsorption capacity of MIP@SiO_2_@QDs and NIP@SiO_2_@QDs for CAP, *M* (g×mol^−1^) denotes the relative molecular mass of CAP, *V* (L) means the volume of solution, *c*_1_ and *c*_2_ (μmol × L^−1^) stand for the CAP concentration in the solution before and after adsorption, and *m* symbolizes the quality of adding MIP@SiO_2_@QDs and NIP@SiO_2_@QDs. 

### 3.6. Optimization of Elution Times Experiment

MIP@SiO_2_@QDs@CAP prepared by adsorption experiment was eluted with a mixture of ethanol and acetonitrile (*v/v*, 8:2). The eluent was collected and the amount of CAP was determined by measuring the CAP in terms of UV absorbance at 278 nm. The above experiment was repeated five times to investigate the elution efficiency with different elution times.

### 3.7. Exploration of Acid and Alkali Resistance of MIP@SiO_2_@QDs

A series of pH buffer solutions (the acetic acid–sodium acetate buffer solution: 0.1 mol·L^−1^, pH 4.0~5.0; the phosphate-buffered solution: 0.1 mol × L^−1^, pH 6.0~10.0; the sodium carbonate–sodium bicarbonate buffer solution: 0.1 mol × L^−1^, pH 11.0~12.0) were prepared. Then, 5.0 mg MIP@SiO_2_@QDs was dissolved in buffer solutions with a different pH in a 10 mL test tube, respectively. In the end, the fluorescence intensity of the well-mixed solutions was measured by fluorescent spectrophotometer.

### 3.8. Selective Experiment

Several structural and functional analogues of CAP (OTC, KAN, THI and MTC) were selected for selective experiments. Under the same conditions, 5.0 mg MIP@SiO_2_@QDs was dissolved in the standard solutions of CAP and its analogues (100.0 μmol × L^−1^, phosphate-buffered solution: 0.1 mol × L^−1^, pH 8.0), separately. After placing 5 h to reach the adsorption equilibrium, the fluorescence intensity of the solutions was detected.

## 4. Conclusions

In summary, through the combination of highly selective MITs and semiconductor materials (QDs) with high fluorescence efficiency, a dual recognition system with a fluorescence quenching of QDs and a specific recognition of MIP for CAP detection was constructed. The molecularly imprinted fluorescent nanomaterial (MIP@SiO_2_@QDs) exhibited wonderful stability and acid-base resistance by the coating of silane layer and the regulation of the cadmium–antimony ratio, while having good specific adsorption performance for CAP. Furthermore, the successful application of MIP@SiO_2_@QDs for CAP detection in lake water indicates that MIP@SiO_2_@QDs has infinite potential for the on-site rapid quantitative analysis of CAP in environment waters.

## Figures and Tables

**Table 1:** Determination results and recoveries of CAP in water samples.

Sample	Found/μmol × L^−1^	Added/μmol × L^−1^	Found/μmol × L^−1^ $\left(\bar{x}\;\pm \;SD,n=3\right)$	Recovery/% $\left(\bar{x}\;\pm \;SD,n=3\right)$
Lake water	0.00	10.00	10.40 ± 0.35	104.0 ± 3.4
	0.00	85.00	85.65 ± 0.65	100.8 ± 0.76
	0.00	150.0	150.78 ± 0.79	100.5 ± 0.52
	0.00	300.0	300.75 ± 0.86	100.2 ± 0.28

**Table 2:** Comparison with other reported methods for detecting CAP.

Methods	Samples	Linear Range (μmol × L^−1^)	LOD (μmol × L^−1^)	Recovery (%)	RSD (%)	Ref.
HPLC	Milk	0.50–10.0	0.20	97.5–104.0	8.5	[8]
CL	Honey	0.50–10.0	0.36	-	-	[9]
ELISA	Sea cucumber	0.38–9.28	0.10	89.0–98.7	11.9	[44]
Electrochemical analysis	Eye drops	2.00–80.0	0.59	98.9–103.0	2.84	[12]
Aptamer Sensor	Milk powder	10.0–1000	5.00	-	-	[14]
MIP-QDs	Lake water	1.00–400	0.35	102.0–104.0	2.06	This work

HPLC—high-performance liquid chromatography; CL—chemiluminescence; ELISA—enzyme-linked immunosorbent assay.

## Data Availability

Not applicable.

## Sample Availability

Samples of the compounds in this article are not available from the authors.
